# Heart in art: cardiovascular diseases in novels, films, and paintings

**DOI:** 10.1186/s13010-020-0086-3

**Published:** 2020-02-13

**Authors:** Ad A. Kaptein, Pim B. van der Meer, Barend W. Florijn, Alexander D. Hilt, Michael Murray, Martin J. Schalij

**Affiliations:** 1 Medical Psychology, Leiden University Medical Centre, Leiden, the Netherlands; 2 Neurology, Leiden University Medical Centre, PO Box 9600, 2300 RC, Leiden, the Netherlands; 3 Nephrology, Leiden University Medical Centre, Leiden, the Netherlands; 4 Cardiology, Leiden University Medical Centre, Leiden, the Netherlands; 5 Psychology, Keele University, Stoke-on-Trent, UK

**Keywords:** Illness perceptions, Cardiovascular diseases, Film, Medical humanities, Novels, Paintings, Self-management

## Abstract

**Background:**

Understanding representations of disease in various art genres provides insights into how patients and health care providers view the diseases. It can also be used to enhance patient care and stimulate patient self-management.

**Methods:**

This paper reviews how cardiovascular diseases are represented in novels, films, and paintings: myocardial infarction, aneurysm, hypertension, stroke, heart transplantation, Marfan’s disease, congestive heart failure. Various search systems and definitions were used to help identify sources of representations of different cardiovascular diseases. The representations of the different diseases were considered separately. The Common Sense Model was used a theoretical model to outline illness perceptions and self-management in the various identified novels, films, and paintings.

**Results:**

Myocardial infarction followed by stroke were the most frequently detailed diseases in all three art genres. This reflects their higher prevalence. Representations ranged from biomedical details through to social and psychological consequences of the diseases.

**Conclusions:**

Artistic representations of cardiovascular diseases reflect cognitions, emotions, and images of prevalent disease. These representations shape views and behaviour of ill and healthy persons regarding heart diseases. As these representations are amenable to change, they deserve further research, which may be instrumental in improving the quality of life of persons struck by cardiovascular diseases. Changing illness perceptions appears to be a method to improve self-management and thereby quality of life in patients with various cardiovascular diseases.

## Background

Illness representations tell viewers and readers about the images of illness – they inform us about the narratives patients construct and communicate. Cardiovascular diseases are the most prevalent cause of morbidity and mortality in most societies. Treatment and management of these conditions draw upon biomedical knowledge. However, social and behavioural factors are also involved in the etiology and course of most if not all these diseases. Smoking tobacco, excess alcohol intake, lack of physical activity, vital exhaustion, and social inequality are examples of these factors, illustrating how illness is more than just disease [1]. Studying ‘the story of the patient’ and employing those stories in understanding and improving how patients make sense of their illness, and studying how novels, films, and painting represent the story of the patient is an extension of this quest [2].

Expressions of disease in various art forms reflect societal views of the image of illness. Von Engelhardt has reviewed how various diseases have been represented in novels [3]; Ostherr has considered their representation in films [4]; while Dequeker has considered their representation in paintings [5]. However, none have specifically considered artistic representations of cardiovascular diseases. In earlier papers we studied how respiratory disorders and lung cancer are depicted in various art forms [6]. In this paper we consider the representation of cardiovascular diseases in novels, films, and paintings.

## Method

A selection of novels, films, and paintings where cardiovascular diseases represent a meaningful and substantial component, was identified using several different approaches:

- The website from New York Columbia University Medical Humanities [www.medhum.med.nyu.edu] was searched, using the search terms ‘myocardial infarction’, ‘aneurysm’, ‘hypertension’, ‘stroke’, ‘heart transplantation’, ‘Marfan’, and ‘congestive heart failure’. These terms were combined with the categories ‘novels’, ‘films’, and ‘paintings’.
- In the NIH PubMed system the following searches were performed: ‘novels AND myocardial infarction’, ‘… AND aneurysm‘, ‘... AND hypertension’, ‘… AND stroke/cardiovascular accident’, ‘… AND heart transplantation’, ‘… AND Marfan’, and ‘… AND congestive heart failure’. These searches were also carried out for ‘film’ and ‘painting’, replacing ‘novels’.
- The work by Von Engelhardt is a major source for researchers studying the way in which literary works discuss diseases [3]. The search terms ‘myocardial infarction’, ‘aneurysm’, ‘hypertension’, ‘stroke’, ‘heart transplantation’, ‘Marfan’, and ‘congestive heart failure’ were checked in the indexes of the five books making up this data base.
- In the Internet Movie Database (IMDb) the following search terms were used: ‘heart attack’, ‘aneurysm’, ‘hypertension’, ‘blood pressure’, ‘stroke’, ‘heart transplantation’, ‘Marfan’, and ‘congestive heart failure’.
- The files of the authors of this paper contain papers on publications in journals in the cardiology category and beyond, where ‘living with cardiovascular diseases and art’ is represented. These papers also are included in our analysis.

With this selective review we intend to examine how various art genres may help in exploring how patients with various cardiovascular diseases give sense to symptoms and signs, and how they manage the illness (‘self-management’). In the field of health humanities this subject of research and clinical care is a major area of attention, given recent developments in health care, such as patient-reported outcomes, quality of life, shared decision making, and value-based health [7].

## Results

The novels, films, and paintings identified were categorised according to seven major diagnostic categories of cardiovascular pathology (Table 1). Each of these is discussed separately.

**Table 1:** Writings (authors), films , and paintings (reviewer ) representing six categories of cardiovascular diseases

Diagnostic category	Writings	Films	Paintings
Myocardial infarction	*Everyman* (Roth) [24] *Own death* (Nádas) [25] *Ooh baby baby* (Jones) [26]	*Dr. Zhivago* [31] *The Godfather* [32] *Something’s gotta give* [33]	*Ket* (de Haas) [34]
Aneurysm	*A study in scarlet (*Doyle) [35]	*-*	*Gidlund* (Bergqvist) [36]
Hypertension	*Everything that rises must converge* (O’Connor) [37]	*Something’s gotta give* [33]	-
Stroke	*Clock without hands* (McCullers) [38] *The loss* (Lenz) [39] *La Toccatina* (Pirandello) [40]	*Amour* [45] *Flawless* [46]	-
Heart transplantation	*Mend the heart (*de Kerangal) [47] *Intruder (*Nancy) [48]	*John Q* [49]	*Calne* [50]
Marfan	*A tale of the ragged mountains (*Poe) [51]	*Mo* [52]	*Schad* (Strauss) [53]
Congestive heart failure	*Dr. Martino* (Faulkner) [54]	*-*	*Boccaccio (Galassi)* [55]

We use a solid theoretical model of living with (chronic) illness, i.e., the Common Sense Model to structure the representation of selected cardiovascular diseases in the three art genres [8]. The model can be summarized below:

symptoms → illness perceptions → coping & self-management → outcome

People perceive symptoms and attach meaning to them. Illness perceptions pertain to cognitions (ideas, views) and emotions about symptoms and signs; coping to how people respond to the challenges an illness poses; self-management to skills (behaviourally, cognitively, emotionally, and socially) that people use to adjust to the illness in their daily lives [8].

### Myocardial infarction

In a systematic review, illness perceptions were shown to be related to outcomes in patients with myocardial infarction: worsening illness perceptions implied poorer quality of life and elevated anxiety and depression [9]. In a study on the effects of cardiac rehabilitation, illness perceptions (i.e., sense of control, perceived duration of the illness, symptom management) predicted perceived effectiveness of cardiac rehabilitation (self-management) programme [10].

### Aneurysm

Aneurysm is only modestly explored in the context of the Common Sense Model. Tomee et al. reported the views of male patients under surveillance for a small abdominal aortic aneurysm. Illness perceptions were assessed with the classic questionnaire for this purpose (IPQ-R). The patients reported great confidence in their health care providers. These perceptions translated into self-management behaviours (e.g., sexuality, life style) [11].

### Hypertension

Spikes et al. report how in Black women in the USA, illness perceptions determined adherence with medication, and thereby with (systolic) blood pressure [12].

### Stroke

In a longitudinal design Groeneveld and colleagues found illness perceptions in a sample of 184 stroke survivors to be associated with physical and mental health outcomes 12 months post stroke [13]. Beliefs of ambulatory people with stroke about using a cane during walking were found to be predictors of cane use; fast walkers perceived use of a cane negatively and did not chose the instrument due to perceptions of stigma [14].

### Heart transplantation

Hofman et al. published a systematic review of smoking after heart transplantation and found that smoking resumption is underestimated in this population; perceived self-control was an illness perception associated with absence of smoking resumption [15]. In adolescents with heart transplantation, qualitative interviews showed how perceptions of life after transplant were associated with quality of life [16].

### Marfan

Connors et al. in a study with semi-structured interviews in individuals with genetic aortic disorders, including Marfan syndrome, found that the inability to incorporate the illness into their life impaired coping and adhering to their physicians’ recommendations, illustrating the importance and relevance of illness perceptions and self-management [17]. Two studies by Peters et al. focused explicitly on illness perceptions and self-management in people with Marfan. Illness perceptions played a major role in shaping everyday life of the patients in every respect [18, 19].

### Congestive heart failure

Timmermans et al. studied illness perceptions in a large sample of patients with heart failure and established associations of those perceptions with health status and poor ICD acceptance [20]. Hundt et al. found illness perceptions to be associated with coping behaviour and conclude that supportive psychological interventions may reduce illness intrusiveness [21] (cf. Lerdal et al.) [22]. Bartlett et al. write about a personalized self-management system. Knowledge and self-management were positively affected by the home-based system [23].

The findings of the empirical studies discussed above are reflected to a considerable extent in works of art. Novels, films, and paintings offer qualitative information on the meaning of the illnesses (i.e., illness perceptions) and on how patients afflicted cope with and self-manage the illness.

### Myocardial infarction

#### Writings

In the novel *Everyman* Philip Roth tells the story of a middle-aged man with a cardiovascular disease history [24]. The protagonist starts suffering from extreme fatigue and breathlessness. Laboratory data indicate “severe occlusion of his major coronary artery (p. 42) … the doctors gave him five grafts” (p. 44). Roth details the implications of the disease – and the illness – not only for the benefit of the patient, but also for the benefit of the nurses, wife, lovers, family members, and doctors. Carotid artery surgery follows as does a “silent heart attack on the posterior wall because of an obstructed graft” (p. 71). Roth tells us that “old age isn’t a battle, it is a massacre”. The protagonist says that “… he was hounded by the sense that he was headed for the end. … he decided to oppose the sense of estrangement brought on by his bodily failing and to enter more vigorously into the world around him” (p. 79). The novel starts with the funeral of the main character; it ends with a carotid endarterectomy, leading to a cardiac arrest.

*Own death* by Péter Nádas is one of the few novels that encompasses the early symptoms, the actual event of a myocardial infarction, its ensuing medical care in a hospital, and the return to the home of the protagonist [25]. The early symptoms are familiar to anyone working in a cardiology setting: “Upon walking I noticed that everything was amiss (p. 11), … I didn’t understand what was going on (p. 17), … I felt deeply nauseous ... (p. 25), … I hadn’t the strength to follow the slight rise of the sidewalk (p. 37), … you don’t understand what is happening, yet you know exactly that this is what they call the sweat of death (p. 55), … waxy, grey complexion (p. 67), … there was no air … (p. 69), … a mighty force pressed against my breastbone and tightened my shoulder blades; it hurt as if I were growing wings” (p. 83). Another quote makes clear that it may not always be easy to extract illness perceptions and self-management from literary text. E.g., “for a long time I didn’t dare leave the house, for it was difficult to take the real existence of things seriously … “(p. 283).

*Ooh baby baby* by Thom Jones is a story of a physician initially denying his cardiac problems but subsequently dying from them [26]. … “For a minute there I thought I was having a heart attack. But when I move around,” he said, breathing heavily, “when I move around – exertion doesn’t exacerbate the pain – it had to be the candy bars … anyhow, I took aspirin …. “(p. 139). Denial is the favorite coping style of this physician.

In the health humanities and the literature & medicine field autopathographies are viewed as not belonging to the scientific corpus [27]. One reason being the sheer number of autopathographies. Another relates to the literary quality which is considered to be rather low by most in the more scientific area of literature & medicine. We, nevertheless, mention a few examples of autopathographies, selected of the basis of at least some (or more) quality. Joan Didion, in *The year of magical thinking*, gives an account of her experiences associated with the sudden death of her husband due to a myocardial infarction (MI) followed by her experiences in the year after his death [28] . Grief is the central concept. Kees van Kooten, a Dutch author and comedian, wrote about his MI (*Long live well-being*) [29], admitting to feeling somewhat “famous and vain” when he was rushed to a hospital in an ambulance with clear signs of the problem, “as if I was on television”. Jan Kott in *The Infarct* writes about his own myocardial infarction: “I was having a heart attack. Until then it was always others who had heart attacks (p. 73) … after the infarct, the heart is constantly present “(p. 83) [30].

#### Films

In *Dr. Zhivago*, the protagonist suffers a myocardial infarction while travelling on a streetcar [31]. Strong emotions about a woman he loves elicit symptoms of angina, with a very quick death soon after.

In *The Godfather*, the protagonist suffers a (fatal) MI while playing with one of his grandsons in the garden [32]. The godfather’s depiction of dying because of a myocardial infarction reflects social images of illness perceptions related to cardiac pathology.

In *Something’s gotta give* a myocardial infarction in the protagonist is precipitated by sexual activity [33]. While the scene is meant in a humorous way, it addresses an important point that prevents taking a reliable history: shame. Applicable to both the physician and the patient. Regularly, one of the parties, or even both, doesn’t feel comfortable discussing sexual issues. Denial of symptoms and erratic adherence, reflecting illness perceptions, are associated with suffering a myocardial infarction in this movie.

#### Paintings

**Fig. 1:**
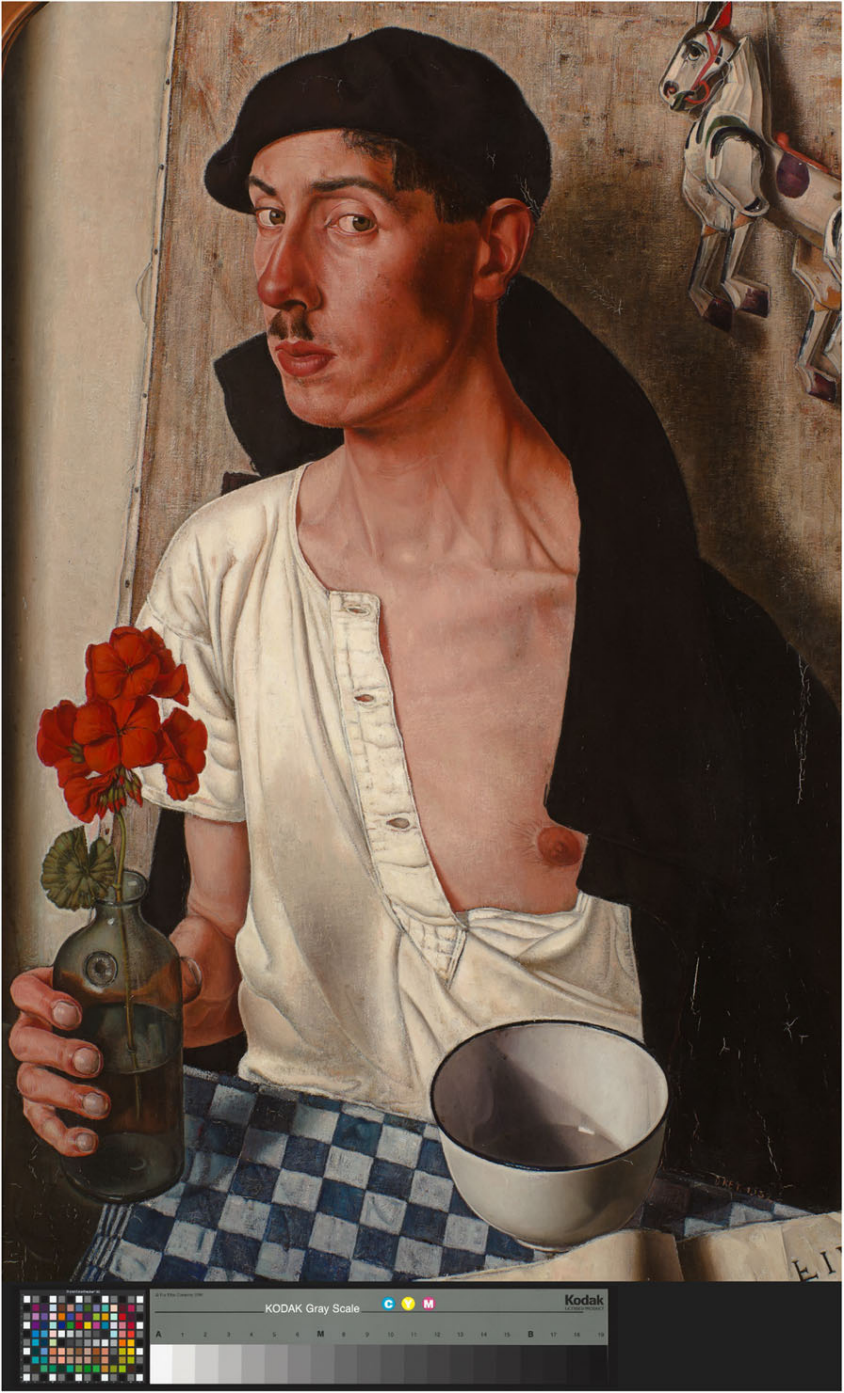
*Self-portrait* (1932) Dick Ket [34]

Dick Ket has painted his cardiovascular pathology in great detail [34]. He suffered from dextrocardia. His self-portrait depicts ‘voussure cardiaque’, nail clubbing, shadow of a heart on his white shirt, anxiety, and the word FIN (Fig. 1). His painting also reflects emotions regarding his cardiac pathology: worry, and attempts to keep control over his pathophysiological situation. Ket is described as being aware of being at risk for sudden cardiac death.

### Aneurysm

#### Writings

In *A study in scarlet*, Conan Doyle (a physician) provides a vivid description of an aneurysm, in his description of a prisoner:

… “I’ve got a good deal to say,” our prisoner said slowly. “… Are you a doctor?”... “Yes, I am,” I answered. “Then put your hand here,” he said, with a smile, motioning with his manacled wrists towards his chest. I did so; and became at once conscious of an extraordinary throbbing and commotion which was going on inside. The walls of his chest seemed to thrill and quiver as a frail building would do inside when some powerful engine was at work. In the silence of the room I could hear a humming and buzzing noise which proceeded from the same source. “Why,” I cried, “you have an aortic aneurism!” “… I went to a doctor last week about it, and he told me that it is bound to burst before many days passed. It has been getting worse for years. I got it from overexposure and underfeeding … “(36; p. 80). Many dimensions of illness perceptions are discernable in this patient’s tale: identity, perceived causes, consequences, timeline [35].

The patient/prisoner dies the next day, due to the rupture of his aorta.

#### Paintings/graphic representation

**Fig. 2:**
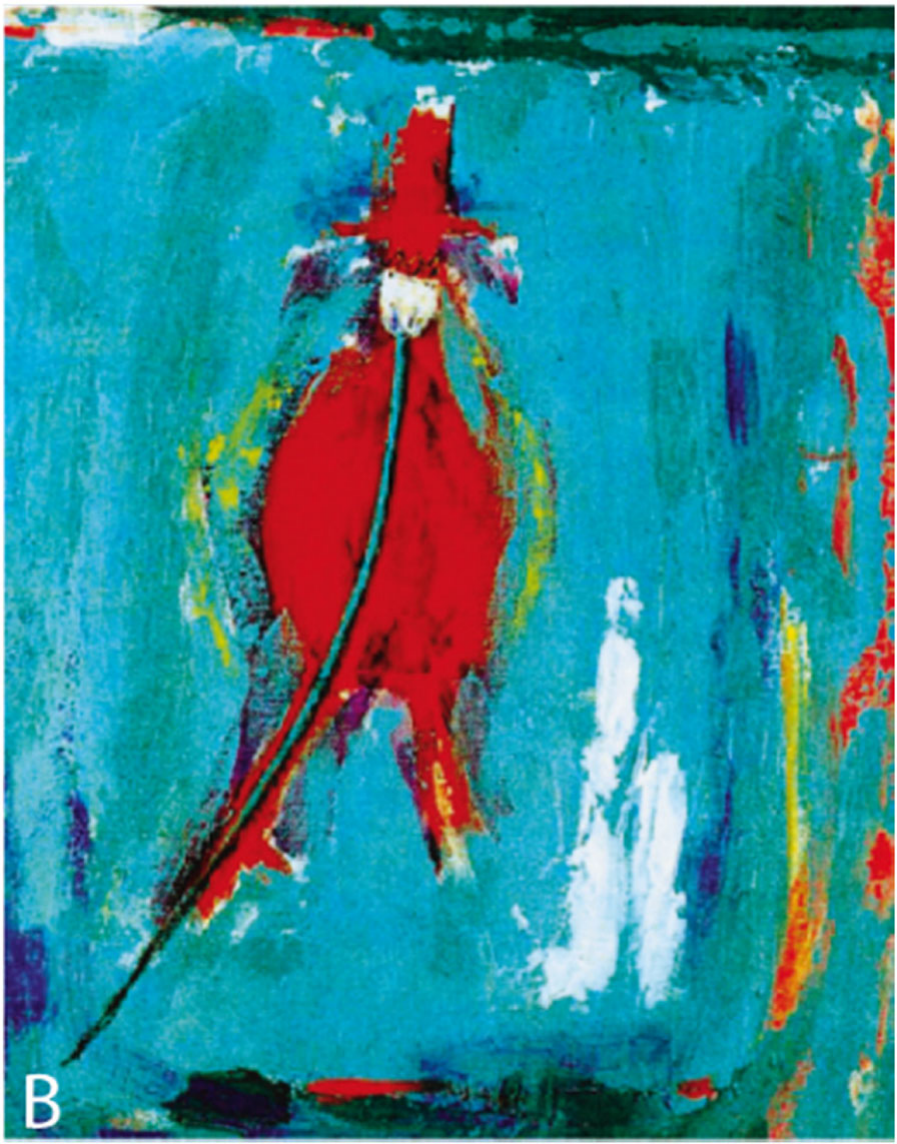
Birgitta Gidlund (in Bergqvist [36]), *Abdominal aortic aneurysm*

Bergqvist, a professor emeritus of cardiology, impressed by ‘the aesthetic beauty of cardiac pathology’, published a paper with a painting, a ceramic object, and a wooden representation of an aortic aneurysm [36] (Fig. 2). The painting is the only representation on canvas of an aneurysm that we were able to locate.

### Hypertension

#### Writings

There appear to be few *novels or writings* where hypertension plays a role. Flannery O’Connor’s’ *Everything that rises must converge* tells the story of a middle-aged woman with hypertension [37]. “Her doctor had told Julian’s mother that she must lose twenty pounds on account of her blood pressure, so on Wednesday night Julian had to take her downtown on the bus for a reducing class at the Y”. (p. 485). The author uses the perception of anger in the mother as the cause for extreme high blood pressure with potential fatal consequences. An event shattering her feeling of self-worth elicits extreme emotional upset, resulting in a stroke. In just 15 pages her slight irritation explodes into a full-blown stroke – and death.

#### Films

When the protagonist in *Something’s gotta give* is admitted to the hospital because of a myocardial infarction, the MD asks him which medication he uses [33]. The protagonist tells the MD he is prescribed atorvastatin and antihypertensive drugs. However, initially he does not dare to confess he took sildenafil, which has a potential dangerous pharmacologic interaction with nitroglycerin the MD has just administered. Denial of symptoms and erratic adherence, reflecting illness perceptions, are associated with suffering a myocardial infarction in this movie.

### Stroke

#### Writings

In *Clock without hands,* Carson McCullers introduces a retired judge as one of the protagonists, with a fairly extensive medical history: insulin dependent diabetes and a stroke [38]. Another central character is Malone [‘I’m alone’], a pharmacist who is diagnosed with leukaemia by the local doctor. The judge and the pharmacist struggle with the effect of their illness on their daily lives. Reinforcing each other’s unhealthy eating and drinking behaviours and non-adherence to medication are a major part of their interaction. “The slide showed it was leukemia. And the blood count showed a terrible increase in leucocytes”. “Leucocytes?”, asked the Judge. “What are they?”. “White blood cells.” “Never heard of them.”” But they’re there”. … “If it was your heart or liver or even your kidneys I could understand your alarm. But an insignificant disorder like too many leucocytes does seem a little far-fetched to me.” (p. 621).

*The loss* by Siegfried Lenz has stroke as its central theme [39]. Situated in post-war Germany, the protagonist suffers a stroke while performing his daily work. The description of onset, course and outcome, and its psychological and social concomitants is vivid and painful:

“It struck him unexpectedly. … He suddenly suffered double vision … he experienced throbbing in his temples. He listened to the loud banging that caused a pressure, a narrow band that laid itself around his head and that caused a pain that he felt the strongest at the back of his eyes. He longed for darkness (p. 10)” […] “he had to throw up, his hand was shaking, his pupils were rigid and wide, his left arm shocked with tremors ... “(p. 27) … he watched his hand in horror, he felt as if it was a strange being, independent and excited, a being that did not acknowledge his control (p. 15) … don’t you see how much I struggle to say the most necessary, what makes you look at me so horrified ... I see the horror and anxiety on your face … (p. 30) … he realized that he suffered the most by knowing that he could not express himself towards another” (p. 133).

The impact of a stroke on the social existence of the sufferer is illustrated in *La Toccatina* [The light touch] by Pirandello. As detailed by Van Haaren et al., the image of stroke some 100 years ago reflects societal views about illness and its consequences [40]. In the paper, the narrative of a twenty-first century patient with a stroke is compared to the one in La Toccatina. The authors maintain that societal norms about desired behaviour determine the objectives of rehabilitation efforts in care for persons who suffered a stroke: from ‘mobility’ in the 19 th century, to ‘communication’ in the twenty-first century.

Several autopathographies on stroke are available as well. Taylor, a neuroanatomist, wrote about her “major haemorrhage, due to an undiagnosed congenital malformation of the blood vessels in my head” (p. 1) [41]. She wrote about the symptoms before, during and long after the stroke. Three decades earlier another medical professional, ‘a 62-year old professor of anatomy’, reported his self-observations and neuro-anatomical considerations after a stroke [42]. His 20- page paper describes motor function, complex functions, such as handwriting, before and after, and other aspects of central nervous function. In Dutch literature two books are noteworthy. Journalist Max Pam wrote about his stroke in *The abyss* including details of his recovery [43]. The other author is very much less fortunate. International chess grand master Jan Hein Donner chose the title *Written after my death* for his autopathography. He died a few years following the stroke [44].

#### Film

Two films depict the functional, psychological, and social consequences of stroke. In *Amour*, the relative stability and happiness of a married couple are destroyed by the stroke that hits the wife [45]. The film presents the viewer with a stroke’s everyday consequences, and eventual sad outcome. Particularly, both the loneliness and isolation brought about by the stroke in both the wife and husband are striking. The female actor playing the victim struck by a stroke in *Amour* impresses in her depiction of the functional effects (paralysed arm, hand), and of the psychological (depressed mood, denial of symptoms), and social consequences (she gets into verbal fights with a nurse). In the second film, *Flawless*, the protagonist is a retired firefighter in New York City, single, who suffered a heart attack - his physician however, talks about a stroke [46]. Robert de Niro plays the victim and shows the consequences: he is unable to open a bottle with medication, he cries when he finds out that he can only walk aided by a tripod and he refuses to attend a party thrown by the other tenants of the (residential) building he lives in. He does, in the end, accept help from one of those tenants – a remedial teacher who is explicitly gay, to the initial horror of the firefighter. Together they end up restoring the victim’s language skills to a considerable extent. The changes in the illness perceptions of the patient, brought about by his neighbor, result in changes in self-management, a theme that illustrates the clinical relevance of studying illness perceptions and self-management.

### Heart transplantation

#### Writings

Two novels are devoted to heart transplantation, both in French. In *Mend the heart* Maylis de Kerangal follows the life of the donor of the heart – a 18-year-old victim of a car crash – and the receiver of the heart, and the roles of a number of persons between donor and receiver [47]. De Kerangal adopts the chronological order of the many phases in the process of the heart transplantation, making the reader aware of the complexity of such a procedure. In the story, evolving over 24 h, a long list of ‘players’ appears: the adolescent who as a consequence of a car accident ends up brain dead and becomes the donor of his heart, his parents who struggle with giving permission for turning off the life support system for their son, helicopter pilots who transport the heart to the recipient’s location, nurses, transplant coordinator, ICU-staff, surgeons, recipient. Psychological, financial, ethical and medical issues are discussed in detail. The storyline in Mend the heart by De Kerangal is unique in the sense that it describes in rather great detail the (illness and treatment) perceptions of the impressive number of participants in the heart transplantation procedure.

In *L'Intrus* (The Intruder), the author J.L. Nancy is the recipient of a heart himself. In a short story format, some 16 pages, the author philosophizes about the meaning of the donor and the donor heart in his life [48]. “… my survival is inscribed in a complex process woven through with strangers and strangeness (p. 5) … it is not that they opened me wide in order to change my heart. It is rather that this gaping open cannot be closed.” (p. 10). He writes about his perceptions regarding receiving a new heart. “My heart was becoming my own foreigner – a stranger precisely because it was inside” (p. 4, English translation); “… it is this myself who becomes my own intruder in all these combined and opposing ways (p. 10) … I am nothing of what I am supposed to be (husband, father, grandfather, friend) unless I remain subsumed within the very general condition of the intruder, of the diverse intruders that at any moment can appear in my place in my relations with, or in the representations of, others” (p. 12).

#### Films

In *John Q* a successful heart transplantation is demonstrated in a desperate father’s child, whose insurance would not cover the transplantation [49]. Therefore the father holds up the hospital and forces them to do the necessary surgical procedure. Although a fictional story, it portrays how a part of American society perceives health care insurers: as evil, and the film puts forward the question whether universal healthcare would be preferable.

#### Paintings

**Fig. 3:**
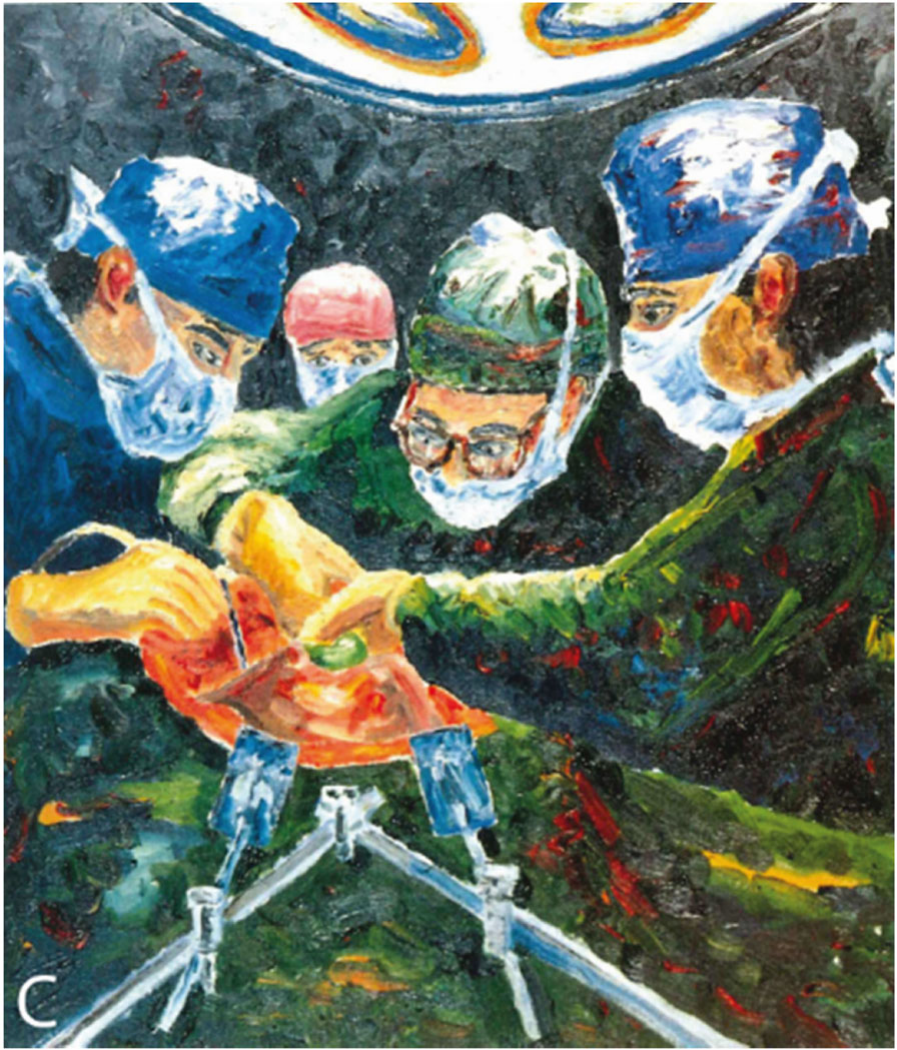
*The moment of truth* [50]

Calne, a transplant surgeon, published a collection of paintings and drawings by himself, documenting the transplantation of hearts, kidney and livers [50] (Fig. 3). His paintings reflect illness perceptions of medical power and optimism about the outcome of highly complex medical procedures.

### Marfan’s disease

#### Writings

Edgar Allan Poe in *A tale of the ragged mountains* tells the story of Mr. Augustus Bedloe – “who was peculiar in his personal appearance” – (p. 208) [51]. A remarkable description follows of someone a contemporary cardiologist would diagnose as a patient suffering from Marfan’s disease:

"He was singularly tall and thin. He stooped much. His limbs were exceedingly long and emaciated. His forehead was broad and low. His complexion was absolutely bloodless. His mouth was large and flexible, and his teeth were more wildly uneven. ... His eyes of a cat ... they were abnormally large and round like those of a cat...their ordinary condition was so totally vapid, filmy and dull, as to convey the idea of the eyes of a long-interred corpse." (p. 1).

It is unclear whether Poe had special knowledge of Marfan’s disease, or whether Poe knew people with the disease. In any case, Poe’s words are unique: they introduce Marfan’s disease into world literature.

#### Films

In *Mo* the protagonist, who is born with clubbed feet, is diagnosed with Marfan’s syndrome in his adolescence [52]. The movie tragically ends with a fatal aortic dissection or rupture in the protagonist, just 2 weeks before the scheduled aortic surgery. The appearance of the protagonist is fairly atypical for Marfan’s syndrome. The protagonist is the smallest of all his friends, while being tall and thin is characteristic for Marfan’s syndrome. This shows other interests sometimes prevail over displaying a certain disease or syndrome as faithfully as possible in movies.

#### Painting

**Fig. 4:**
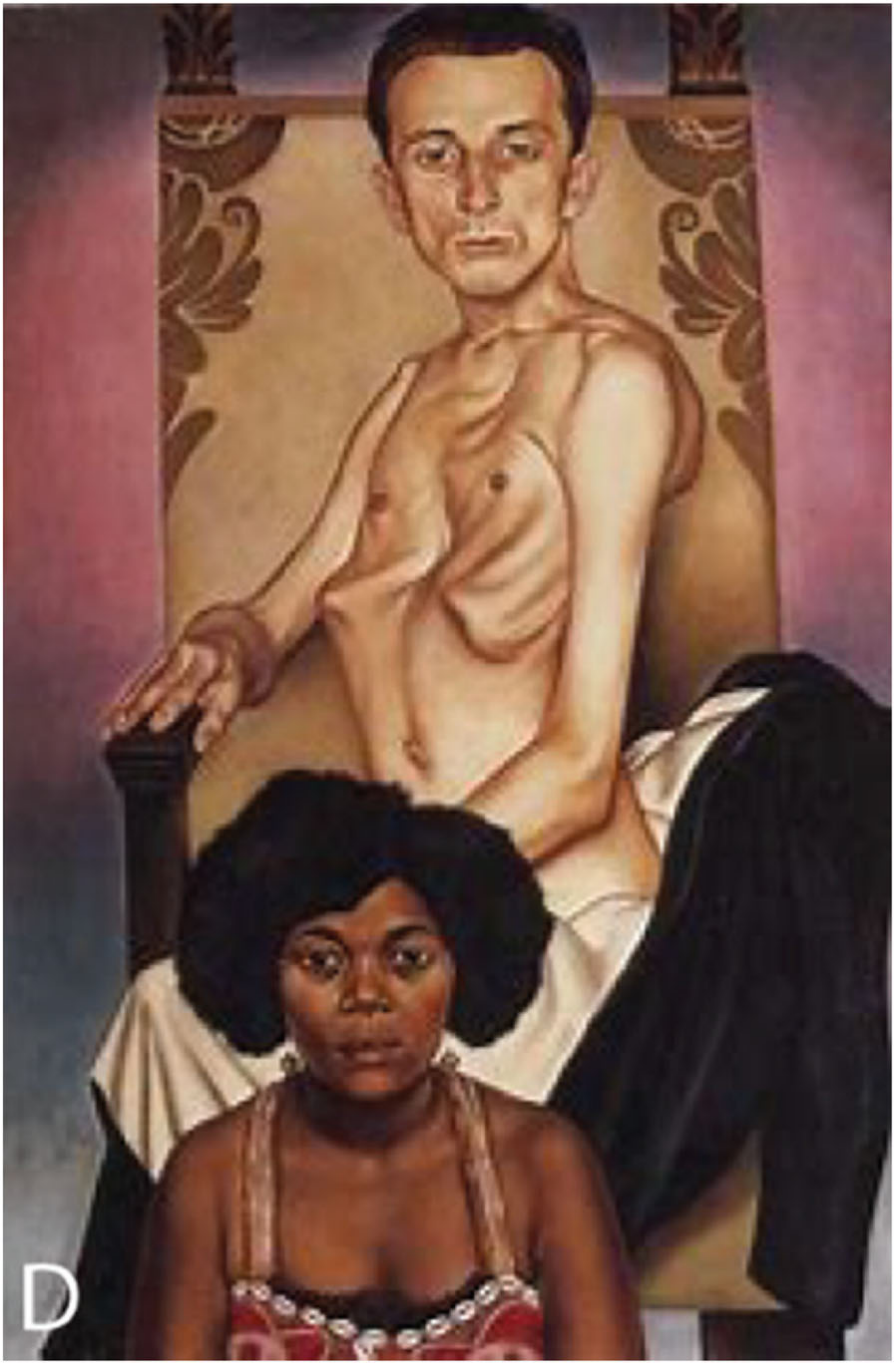
*Agosta der Flügelmensch und Rascha die schwarze Taube*, 1929, painted by Christian Schad (1894–1982) [53]

The painting *Agosta der Flügelmensch und Rascha die schwarze Taube* by Christan Schad is a spectacular depiction of Marfan’s disease (Fig. 4) [53].

### Congestive heart failure

#### Writings

We identified a novel (Dr. Martino by William Faulkner [54]) and a painting of Bocaccio, who apparently suffered from congestive heart failure.

“It’s his heart, … he has to be careful. He had to give up his practice and everything. … Each summer I think it will be the last time; that’ we shan’t see him again. But each May I get the message from him, the reservation.” (p. 169) - Dr. Martino by William Faulkner describes the illness history of a physician in the South of the USA, who suffers from congestive heart failure.

#### Painting

A recent study on the medical history of Bocaccio, author of the Decameron, maintains that the author suffered heart failure. The paper contains a painting of Bocaccio, suggestive, according to the authors, of heart failure [55].

## Discussion

Cardiovascular disorders are the major cause of mortality in highly industrialized societies. The morbidity, societal and financial impact are also major. These considerations contrast remarkably with the rather modest attention to these diseases in various art genres. It is not immediately clear what the background of this finding might be. In oncology, a somewhat different situation seems to be observable: various scientific journals devote substantial attention to the representation of cancer and suffering from cancer in novels, film, painting, and music. General medical journals (e.g., British Medical Journal (*BMJ),* Journal of the American Medical Association *(JAMA)*, Lancet) have niches on ‘patient narratives’. No such journal exists in the cardiology domain.

The overall picture that seems to be discernible from the novels, films and paintings that we discussed here pertains to the dramatic and intense immediate symptoms and consequences of myocardial infarction, the long term consequences of stroke, the relatively low level of impact of hypertension, and the very major concomitants of heart transplantation and the somewhat mysterious phenomena surrounding Marfan’s disease. These observations are based on the novels that we identified. The films where cardiovascular diseases play a prominent role corroborate this impression. Given the very few paintings where cardiovascular diseases are depicted, it is somewhat difficult to draw too strong conclusions.

The search systems used would deserve further development and refinement. We were unable to find a search system that produced unequivocally a set of novels – in major languages – on, for instance, myocardial infarction.

The number of diagnostic categories within cardiology that can be discussed in one paper is limited. We limited ourselves to seven diagnostic categories within cardiology, therefore. With three art genres, this implies touching upon a total of 21 novels, films and paintings – we hope that our paper encourages future researchers to refine and expand our review.

Our review of the way how three art genres offer a picture of the representation of various cardiological diseases does show the major impact of the diseases, their relative long-term chronic nature, and the need for patients to adjust. Further exploration of the way in which various art genres depict the different categories of chronic diseases, and of the way in which forms of therapeutic applications of writing, filming and painting may impact on patient reported outcome measures (PROMs) in patients and their caregivers and health care providers, seem worthwhile research areas [56].

Our select review of how patients with various cardiovascular disorders make sense of their illness, as represented in novels, films and paintings, points at starting points for clinical interventions. After all, addressing maladaptive ways of making sense of the illness, which lead to maladaptive coping and self-management and poor outcome should result in more adaptive coping, self-management and a positive outcome. Empirical studies do indeed support this line of reasoning. Broadbent et al. in their systematic review and meta-analysis of illness perceptions report that illness perceptions “were able to predict some outcomes up to one-year follow-up” ([57] p. 1361). This pertained to a wide range of illnesses, strengthening the validity and clinical usefulness of the concept of illness perceptions. As an example outside cardiovascular medicine, the study by Chilcot et al. demonstrated how changes in illness-related cognitions mediate improvements in symptoms and disability in persons with irritable bowel syndrome [58].

The almost prototypical intervention study in the area of patients with cardiovascular disease and illness perceptions is the study by Petrie et al. [59]. In a randomized control design, patients in the experimental group were offered a three-part intervention, whereas patients in the control group received standard medical care. The intervention (a) explored illness perceptions, (b) addressed maladaptive thoughts and behaviour patterns, and (c) helped patients substitute maladaptive thoughts, cognitions and emotions with more adaptive, constructive ones. The results showed significant improvements in resumption to work, fewer symptoms. In a summary paper, Petrie and Weinman conclude that “patients do not usually spontaneously disclose their illness beliefs in the consultation” [60]. Therefore, assessing these beliefs or perceptions seems a wise investment. Valid, reliable and concise questionnaires are available in the literature (e.g. [57]). Busy clinicians may involve nurses to help collect data on illness perceptions [56]. The authors further conclude that “interventions to change illness perceptions can reduce disability and improve functioning” (p. 539 [60]). Here also, nurses or psychologists may be professionals who are experts in exploring, addressing and helping to change illness perceptions. A recent paper in Heart [61] and a recent paper in Cardiovasc Diagn Ther [62] summarize it all: “Heart and mind: behavioural cardiology demystified for the clinician”, and “Towards a narrative cardiology: exploring, holding and re-presenting narratives of heart disease”, respectively.

## Data Availability

The dataset used and/or analysed during the current study are available from the corresponding author on reasonable requires.

## Abbreviations

BMJ: British Medical Journal
IMDb: Internet Movie Database
JAMA: Journal of the American Medical Association
MI: Myocardial infarction
PROMs: Patient reported outcome measures
